# Humoral immune response and delayed-type hypersensitivity in rabbits infected with *Trypanosoma equiperdum*

**DOI:** 10.1038/s41598-020-71992-x

**Published:** 2020-09-10

**Authors:** Tiziana Di Febo, Ivanka Krasteva, Barbara Bonfini, Manuela Tittarelli, Osvaldo Matteucci, Gianluca Orsini, Emanuela Rossi, Michele Podaliri Vulpiani, Diamante Rodomonti, Luigi Iannetti, Mirella Luciani

**Affiliations:** Istituto Zooprofilattico Sperimentale dell’Abruzzo e del Molise “G. Caporale”, Via Campo Boario, 64100 Teramo, Italy

**Keywords:** Immunology, Microbiology

## Abstract

*Trypanosoma equiperdum* is the causative agent of dourine, a parasitic venereal disease of equids. In this work, rabbits were infected with *T. equiperdum* strain OVI; serological tests (complement fixation test, ELISA and immunoblotting), used for the diagnosis of dourine in horses, were applied to study rabbit humoral immune response and to characterise *T. equiperdum* antigen pattern recognised by antibodies from infected rabbits. Moreover a protein extract of *T. equiperdum* strain OVI was produced and tested in skin tests on infected rabbits to detect the cell-mediated response induced by *T. equiperdum*, in order to evaluate its use in the field diagnosis of dourine. Sera of infected rabbits recognized in immunoblotting *Trypanosoma* protein bands with molecular weight below 37 kDa, providing a serological response comparable with that already observed in dourine infected horses. Moreover the trypanosome protein extract was capable to produce in vivo delayed-type hypersensitivity (DHT Type IV) in rabbits and proved itself to be non-toxic and non-sensitizing.

## Introduction

Dourine is a sexually transmitted disease affecting equids and caused by the protozoan *Trypanosoma equiperdum* (Kinetoplastida, subgenus *Trypanozoon*). Other members of the subgenus are *T. evansi*, the causative agent of surra in a wide range of mammals (horses, cattle, goats, buffalos, dogs, camel, deer, wild pigs, capybaras), and *T. brucei*, the causative agent of sleeping sickness in humans. *T. brucei* affects also domestic (cattle, pigs, small ruminants) and wild animals^1^.

In contrast with other trypanosomes, *T. equiperdum* is not transmitted by an invertebrate vector and it is primarily a tissue parasite that is rarely detected in the blood. Dourine is sexually transmitted; transmission by artificial insemination has not been confirmed, but it could potentially occur since *T. equiperdum* is present in the seminal fluid and in the genital tissues. The average mortality in horses is about 50%; donkeys and mules are more resistant and may remain unapparent carriers. To date, infected equids are the only known reservoir of the parasite^2,3^. No vaccines for dourine are available and pharmaceutical therapy is not recommended; the eradication strategy currently applied imposes slaughtering of seropositive animals^4^. This policy, however, is not economically feasible to be applied in developing countries, where horses are extensively used for transport and agriculture^5^.

Dourine is endemic in Africa and South America^3^ and has been also reported in Mongolia and Kazakhstan^6^. Most countries where dourine and other trypanosomoses, such as surra, are endemic do not regularly notify these diseases, so their spatial distribution remain not well known^7^. Recent outbreaks of trypanosomosis in Europe, as dourine in Italy^8^ and surra in Grand Canaria, Spain and France^9,10^ caused, respectively, by imported infected horses and camels, highlight the risk of importation of equine trypanosomosis into non-endemic countries. In spite of important economic losses caused by equine trypanosomoses, they can still be considered as neglected diseases^11^. Epidemiological investigations highlighted that knowledge of pathogenesis of dourine and host–pathogen interactions is still limited. Moreover the lack of vaccines, the inability of drugs to cure the neurological stage of the disease, and the limitations of current diagnostics make more difficult the control of the disease^7,12^. In addition, differential diagnosis is extremely difficult in geographical areas where *T. equiperdum* coexists with *T. evansi*, because the two parasites are genetically and antigenically related and the clinical outcome of the two diseases in horses is very similar^3^.

A better comprehension of the host immune response following infection could allow to identify targets that could improve the diagnosis of the disease. Despite the availability of in silico models or the development of alternative methods, such as cellular cultures that aim to reproduce tissues and organs, animal experimentation is still necessary to understand the pathogenesis of diseases and to develop and evaluate new therapeutic strategies^13,14^. *T. equiperdum* is able to infect rabbits, showing that this species could be used in the research on dourine^3,15,16^ and other trypanosomoses. Small mammals (rodents and lagomorphs), are preferred for laboratory research instead of other mammals as horses, cattle and sheep, due to their lower maintenance costs, shorter reproduction time and availability of transgenic models. Moreover, small mammals could be useful for the study of diseases that affect larger mammals.

In this work, rabbits were experimentally infected with *T. equiperdum* strain OVI and their humoral response was studied in vivo using serological tests (complement fixation test, ELISA and immunoblotting) currently used for the diagnosis of dourine in horses.

Moreover, a protein extract of *T. equiperdum* strain OVI was produced and used as skin test antigen on rabbits in order to evaluate its performances and its safety. The skin test antigen could be used in the field diagnosis of dourine in horses, in particular in endemic areas, just as brucellin and tuberculin are used in the diagnosis of, respectively, brucellosis and tuberculosis in cattle.

## Materials and methods

### Animal experimentation

Animal experimentation was carried out in compliance with Italian national law (Legislative Decree 26/2014)^17^ implementing Directive 2010/63/EU of the Council of the European Communities on the protection of animals used for scientific purposes^18^. Ethical approval was obtained from the Italian Ministry of Health (Protocol Number 511/2015-PR; DGSAF 11030-A of the 28/04/2015 integrated by the DGSAF 0019112-P of the 08/08/2016, ex Lgs. D. 26/2014, art. 31).

Healthy adult immunocompetent male rabbits, weighting 2.5–3.0 kg, and healthy female guinea pigs, weighting 250 g each, were used. In particular, 18 rabbits were experimentally infected with *T. equiperdum*, 9 rabbits were used as negative controls, 5 rabbits and 2 guinea pigs were used to assess toxicity and sensitization effects of the skin test antigen.

Animals were kept in an enriched environment, considering the needs of behavioural biology, and were caged individually but ensuring eye contact. Animals were maintained in a conventional facility with 12-h light/dark cycle, at 15 ± 5 °C for rabbits and at 20 ± 4 °C for guinea pigs; free access to regular food and tap water were allowed. Social and food enrichment were guaranteed^19^.

### Rabbit experimental infection

*T. equiperdum* strain, provided by Onderstepoort Veterinary Institute, Pretoria, South Africa (OVI *T. equiperdum*), was produced in rats according to the World Organisation for Animal Health (2018)^3^, and stored at − 80 °C until use. Nine rabbits were infected intrascrotally^2,15^ with cryopreserved *T. equiperdum* in order to amplify the parasite and to adapt it to the rabbit. When a marked scrotal edema appeared, rabbits were sacrificed; then the scrotum of each rabbit was removed and the edematous material was taken, homogenized and diluted in 0.01 M phosphate-buffered saline, pH 7.5 (PBS), to get 10^4^ live trypanosomes/ml. Trypanosomes were counted by Bürker counting chamber. This homogenized antigen was inoculated intrascrotally in other 9 rabbits (1 ml for each animal). Blood samples of infected rabbits were collected from the marginal ear vein, after applying local anaesthesia, to verify the presence of antibodies *versus T. equiperdum* before the infection (T0) and at times 7, 14, 21, 28 and 35 days post infection (T7, T14, T21, T28 and T35). As negative control, nine rabbits were inoculated with PBS only and blood samples were taken at time from T0 to T35. Sera were stored at – 20 °C until use. The second group of 9 inoculated rabbits and negative controls were used also to test the skin test antigen.

### Serological tests for the control of rabbit infection

Complement fixation test (CFT) was performed according to the method described for equids in the OIE Manual of Diagnostic Tests and Vaccines for Terrestrial Animals^3^. Briefly, the antigen was produced in rats using the OVI *T. equiperdum*, according to the OIE Manual^3^. Sera were diluted five-times with veronal buffered saline, and those showing more than 50% of fixation level at the dilution 1:5 were considered as positive and tested again to the end point using twofold dilutions.

Indirect-ELISA (i-ELISA) was performed according to Bonfini et al.^20^. Briefly, rabbit sera were diluted 1:100 in PBS containing 0.05% Tween 20 and 0.1% BSA (dilution buffer); as secondary antibody, a peroxidase-conjugated goat Anti-Rabbit IgG (H + L) (Bio-Rad, Hercules, CA, USA) diluted 1:30,000 in dilution buffer was used. Results were expressed as percentage of positivity (PP), calculated using the following formula:

$$ {\text{PP}} = \left( {{\text{OD}}\,{\text{Sample}}{-}{\text{OD}}\;{\text{Negative}}\;{\text{Control}}} \right)/\left( {{\text{OD}}\;{\text{Positive}}\;{\text{Control}}{-}{\text{OD}}\;{\text{Negative}}\;{\text{Control}}} \right) \times 100 $$

As positive and negative controls, a lyophilized serum obtained from a rabbit experimentally infected with OVI *T. equiperdum*^15^ and the serum of a healthy rabbit were used. The cut-off value of the ELISA was calculated by adding 4 times the standard deviation to the mean PP value of sera collected from the 9 rabbits in the control group at times from T0 to T35^21^. Sera showing a PP value greater than the cut-off value were considered positive.

The immunoblotting assay was performed according to Luciani et al.^22^, using rabbit sera diluted 1:5,000 and the peroxidase-conjugated goat Anti-Rabbit IgG (H + L) (Bio-Rad) diluted 1:3,000. Image acquisition was carried out with Chemidoc MP (Bio-Rad) using Image Lab Software version 4.0.1 (Bio-Rad). Sera reacting with bands with molecular weight (MW) equal or less than 37 kDa were considered as positive for *Trypanosoma* antibodies^22,23^.

### Skin test

The antigen was produced from blood collected from rats that had been experimentally infected with OVI *T. equiperdum*^3^ and containing approximately 90% live trypanosomes, as assessed by a Bürker counting chamber. After washing with PBS at 2000 rpm for 20 min at 4 °C, the supernatant was discarded and the buffy coat containing trypanosomes was collected and, after further washings to remove red cells, the pellet was collected, resuspended in PBS and lysed by freeze/thawing cycles in liquid nitrogen. The lysed cells were filtered through 0.8 µm, 0.45 µm and 0.22 µm filters (Millipore, Carrigtwohill, Co. Cork, Ireland). The protein concentration of the antigen was determined using the BCA Protein Assay Kit (Thermo Scientific, Rockford, IL, USA) and adjusted to 1.4–2.0 mg/ml with PBS.

Six weeks after infection with OVI *T. equiperdum*, the 9 infected rabbits and the negative controls were shaved at two rectangular areas of about 3.0 × 9.0 cm on both flanks. Each of the two areas was divided into 3 equal squares. *T. equiperdum* skin test antigen (0.2 ml) was inoculated intradermally into 3 squares according to Latin square design and physiological solution (0.2 ml) into the remaining 3 squares as control. Skin reactions (diameters of erythema) were measured 24 h after antigen injection. The reaction was considered positive if an erythema of 8–25 mm of diameter was present.

### Evaluation of toxicity and sensitization effects of the skin test antigen

Toxicity and sensitization effects of the skin test antigen were evaluated using, respectively, guinea pigs and rabbits.

Two guinea pigs were inoculated subcutaneously with 0.5 ml of *T. equiperdum* skin test antigen and observed for 7 days to evaluate toxicity effects (body weight loss, systemic effects and behavioural variations).

Six rabbits divided into 2 groups (test group and control group) were used to evaluate skin sensitization effects of the antigen. Three rabbits (test group) were inoculated intradermally 3 times at 5-day intervals with 0.1 ml of *T. equiperdum* skin test antigen. Twenty days after the third inoculation, another dose was injected into the same rabbits and into other 3 rabbits, previously not-inoculated with *T. equiperdum* skin test antigen (control group). Animals (test group and control group) were observed from the first inoculation until the end of the experiment (48 h after the last inoculation) to check for signs of sensitization (erythema and/or edema) at the point of injection.

### Statistical analyses

Statistical analysis was performed with the calculation of the percentage of reactor animals (observed or presumed) and the relative uncertainty, according to a Bayesian approach and using a Beta distribution, at 95% confidence interval, considering the percentage of rabbits that gave a skin test positive reaction, and taking into consideration two types of reactions: rabbits that showed positive reactions in all the skin treated areas and rabbits that showed positive reaction in at least one skin treated area.

## Results

### Serological tests for the control of rabbit infection

All the nine rabbits infected with OVI *T. equiperdum* became positive after 7 days from inoculation (T7) (CFT titres ≥ 1:40) and maintained high antibody titers (CFT titres ≥ 1:320) for the entire duration of the experiment (Table 1). The control animals remained negative throughout the experiment.

**Table 1:** CFT antibody titres of rabbits infected with OVI *T. equiperdum.*

Days post inoculation	CFT titres								
	Rabbit #1	Rabbit #2	Rabbit #3	Rabbit #4	Rabbit #5	Rabbit #6	Rabbit #7	Rabbit #8	Rabbit #9
0	Neg	Neg	Neg	Neg	Neg	Neg	Neg	Neg	Neg
7	1:160	1:160	1:160	1:40	1:160	1:160	1:160	1:160	1:80
14	1:320	1:320	1:320	1:320	1:320	1:640	1:640	1:640	1:1,280
21	1:320	1:320	1:320	1:320	1:320	1:320	1:320	1:1,280	1:320
28	1:640	1:640	1:640	1:1,280	1:640	1:320	1:640	1:640	1:640
35	1:640	1:640	1:160	1:640	1:640	1:160	1:640	1:320	1:320

**Figure 1:**
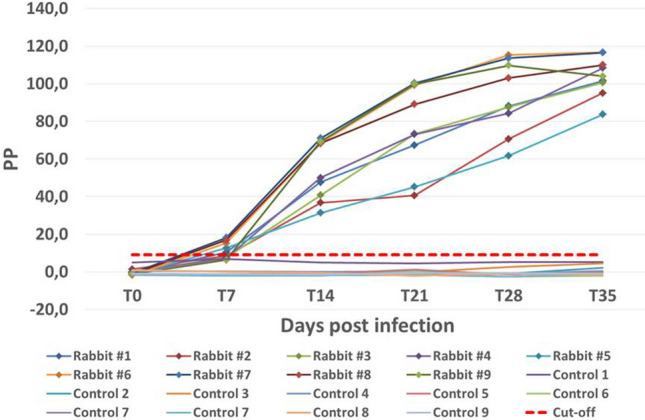
Indirect-ELISA: percentage of positivity (PP) values of OVI *T. equiperdum* infected and not infected (control group) rabbits.

Indirect-ELISA PP values of infected rabbits ranged from 6.3 to 18.2% at 7 days post inoculation (T7) and gradually increased till 83.7–116.7% at time T35. Calculated cut-off was 9.2%; according to this value, at time T7 rabbits #1, #5, #6, #7 and #8 tested positive and rabbits #2, #3, #4 and 9 tested negative, while at time T14 all the rabbits tested positive. PP values for the rabbits of the control group were less than 6.9% throughout the experiment (Fig. 1).

**Figure 2:**
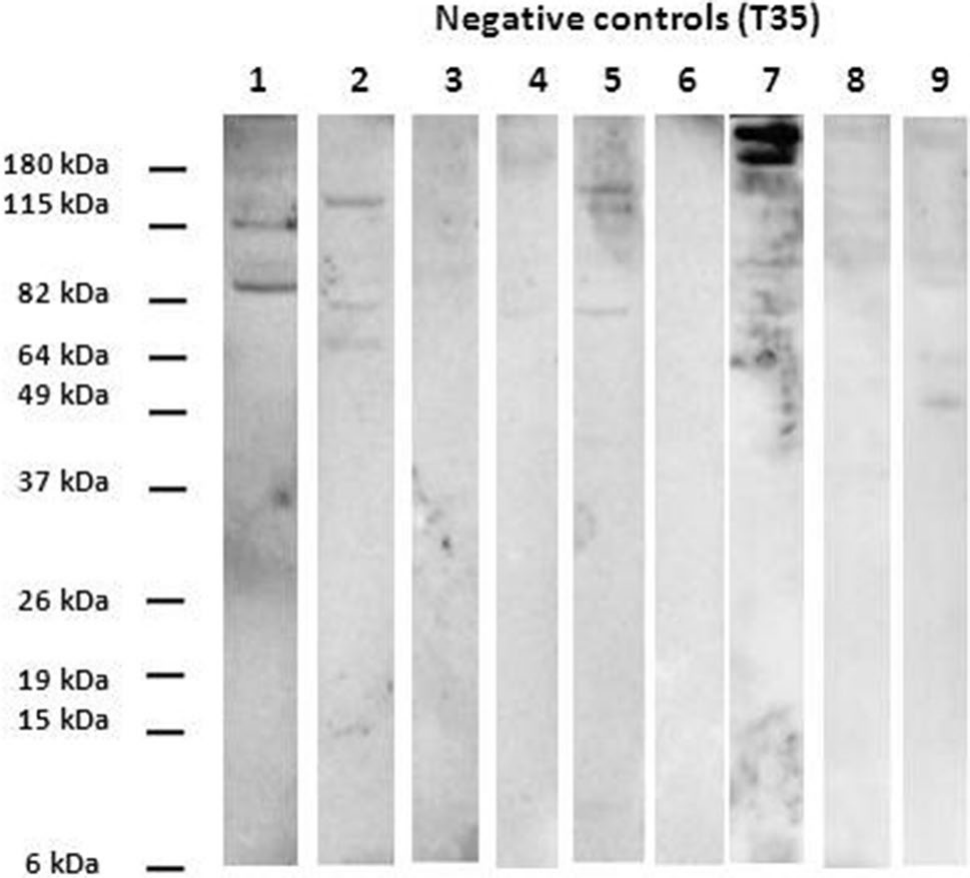
Immunoblotting results of rabbits not infected with OVI *T. equiperdum* (control group) at T35 post infection. Molecular weight marker: BenchMark Pre-Stained Protein Ladder (Thermo Fisher Scientific).

**Figure 3:**
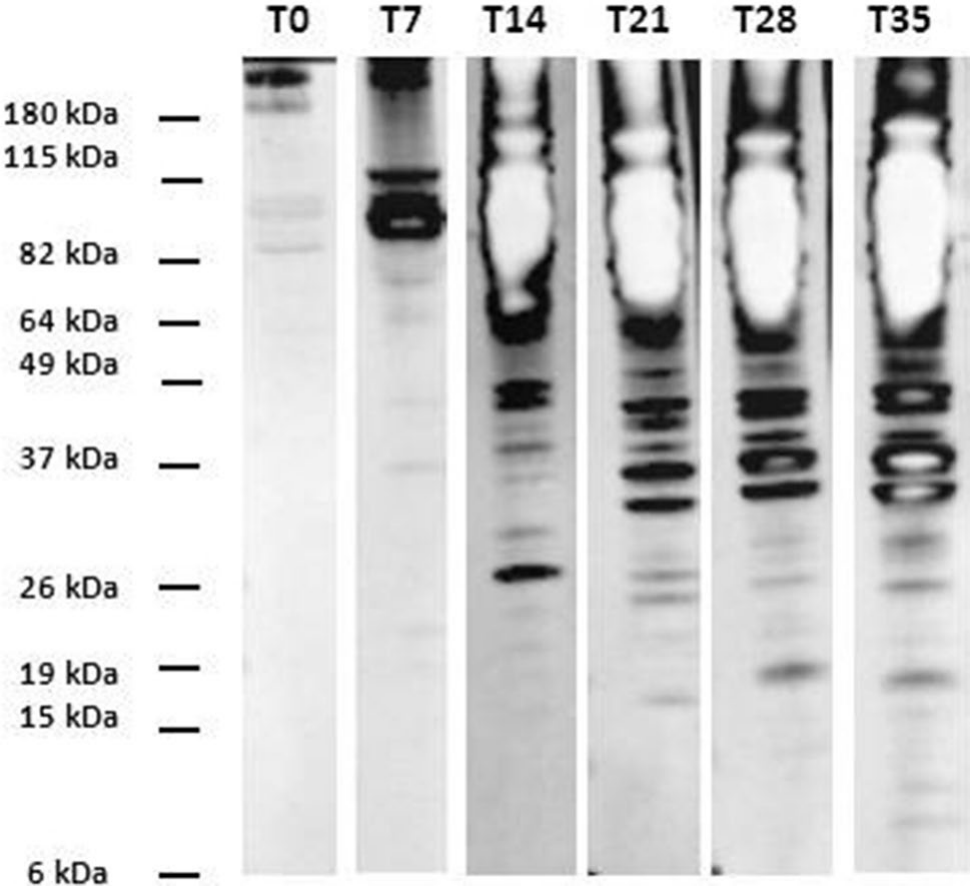
Immunoblotting results of one of the rabbits infected with OVI *T. equiperdum*. Molecular weight marker: BenchMark Pre-Stained Protein Ladder (Thermo Fisher Scientific).

Immunoblotting results showed that rabbit serum antibodies from the control group at times from T0 to T35 identified bands with MW ranging from 40 to 180 kDa (Fig. 2). On the contrary, starting from time T7, antibodies in the sera of infected rabbits showed an increasing reaction with *Trypanosoma* proteins, and in particular with low MW proteins (6–37 kDa) (Fig. 3). More in detail, rabbits #3, #4, #5 and #7 tested negative at T7 but positive at T14; the other 5 rabbits (#1, #2, #6, #8, #9) tested positive at both T7 and T14. At time T7 rabbits #1 and #2 reacted with a weak band of 37 kDa, rabbit #6 reacted with a weak band of 26 kDa, rabbit #8 with bands of 37, 24 and 20 kDa and rabbit #9 with bands of 37, 25, 20 and 15 kDa (images not shown).

The gradual increase in the number and intensity of protein bands recognized by rabbit positive sera during the time, has been already described for horse positive sera in Luciani et al.^22,23^.

Results of the three serological tests of infected rabbits at times T0, T7 and T14 were summarized in Table 2.

**Table 2:** Results of CFT, i-ELISA and immunoblotting of OVI *T. equiperdum* infected rabbits at times 0, 7 and 14 days p.i.

Rabbit	CFT			i-ELISA			Immunoblotting		
	T0	T7	T14	T0	T7	T14	T0	T7	T14
#1	Neg	Pos	Pos	Neg	Pos	Pos	Neg	Pos	Pos
#2	Neg	Pos	Pos	Neg	Neg	Pos	Neg	Pos	Pos
#3	Neg	Pos	Pos	Neg	Neg	Pos	Neg	Neg	Pos
#4	Neg	Pos	Pos	Neg	Neg	Pos	Neg	Neg	Pos
#5	Neg	Pos	Pos	Neg	Pos	Pos	Neg	Neg	Pos
#6	Neg	Pos	Pos	Neg	Pos	Pos	Neg	Pos	Pos
#7	Neg	Pos	Pos	Neg	Pos	Pos	Neg	Neg	Pos
#8	Neg	Pos	Pos	Neg	Pos	Pos	Neg	Pos	Pos
#9	Neg	Pos	Pos	Neg	Neg	Pos	Neg	Pos	Pos

At times from 21 to 35 days p.i., rabbits tested positive at both CFT and immunoblotting, so results are not included in the table.

### Skin test

**Figure 4:**
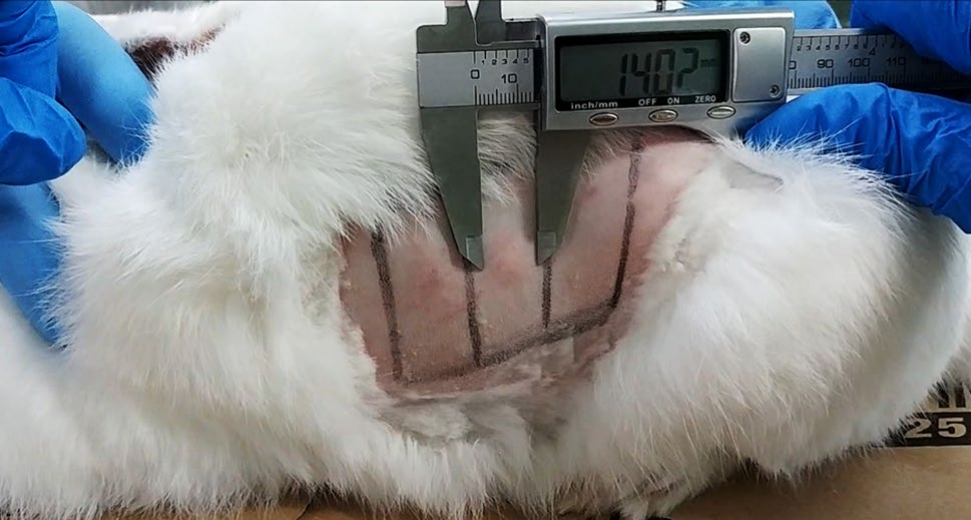
Skin reactions observed 24 h after OVI *T. equiperdum* protein extract injection (infected rabbit #8).

Skin reactions to *T. equiperdum* skin test antigen were observed in 8 out of 9 infected rabbits (Fig. 4). In particular, a positive reaction (diameters of erythema of 8–25 mm) was observed in five rabbits in all the three treated skin areas; in two rabbits, in two out of three treated areas and in one rabbit, in one out of three treated areas. Negative reaction in the three treated skin areas was observed in one rabbit. Moreover, negative reaction was observed in the skin negative control areas.

Statistically significant differences were observed between negative controls and rabbits that showed positive reactions in all the three treated skin areas or in at least one treated skin area (Table 3).

**Table 3:** Calculation of the percentage of reactor animals and the relative uncertainty for rabbits that showed positive reactions in the skin treated areas.

	N. rabbits	N. rabbits with positive skin test reaction	% rabbits with positive skin test reaction	Uncertainty	
				L.C.I. 95%	U.C.I. 95%
Physiological solution	9	0	0%	0%	25.9%
Skin test antigen	9	8^a^	89.0%^a^	55.5%^a^	97.5%^a^
		5^b^	56.0%^b^	26.2%^b^	81.3%^b^

^a^ Rabbits that showed positive reaction in at least one skin treated area.

^b^Rabbits that showed positive reactions in all the skin treated areas.

### Evaluation of toxicity and sensitization effects of the skin test antigen

No clinical signs, changes in body weight or behavioural variations were observed in the two guinea pigs inoculated subcutaneously with *T. equiperdum* skin test antigen.

No dermal responses as erythema and/or oedema were found in rabbits of both test and control group from the first inoculation until the end of the experiment.

## Discussion

Diagnosis of dourine depends on the identification of clinical signs and lesions, the isolation of the parasite and the use of serological tests. The first ones can be similar to those caused by other diseases, such as surra, and differential diagnosis and epidemiological investigations are needed. *T. equiperdum* is present in the blood and tissue fluids of infected horses in small numbers, so its identification is difficult. Moreover, *T. equiperdum* is morphologically very similar to *T. evansi*^3,11^.

Genetic markers for the differential diagnosis between dourine and surra are under evaluation; to date no *T. equiperdum*-specific PCR method is available^3^.

Phylogenetic analysis has suggested an evolution of *T. equiperdum* and *T. evansi* from *T. brucei brucei*; for this reason, and considering the high degree of similarity among the three trypanosomes, some authors have proposed to consider *T. equiperdum* and *T. evansi* as subspecies of *T. brucei* (*T. brucei equiperdum* and *T. b. evansi*)^24,25^.

OIE-prescribed serological tests for dourine are CFT and indirect fluorescent antibody test (IFAT); the latter is currently used to confirm infection or resolve inconclusive CFT results. Other serological tests used in the diagnosis of dourine are ELISA, radioimmunoassay, counter immunoelectrophoresis, agar gel immunodiffusion (AGID), immunoblotting and a card agglutination test^3^. These tests are not specific for *T. equiperdum*, due to antigenic similarities among *T. equiperdum*, *T. evansi* and other *Trypanosoma* species^3,26^.

Three serological tests, developed for the diagnosis of dourine in horses, were applied in the present study to evaluate the humoral response of OVI *T. equiperdum* infected rabbits and to compare the results with the horse humoral response. Infected animals tested positive for anti-*Trypanosoma* antibodies at CFT after 7 days post infection (p.i.) and at i-ELISA and immunoblotting at times between 7 and 14 days p.i.. Moulton et al.^16^ and Hiepe et al.^27^ observed an increase in antibody titres after, respectively, 7 and 9 days p.i. in rabbits infected with *T. equiperdum*. In another work of Bishop et al.^28^, antibodies versus* T. equiperdum* were detected after 14 days p.i. in experimentally infected rabbits and horses. According to Luciani et al.^22^ a mare, experimentally infected by tranfusion of blood collected from dourine naturally infected horses (2011 Italian outbreak), became positive at CFT and immunoblotting after 14 days post infection (p.i.), and tested positive also at IFAT 3 days later (17 days p.i.). Hébert et al.^29^ carried out experimentally infections on horses to validate a model for assessing drug efficacy against *T. equiperdum* infection: 9 horses, intravenously inoculated with OVI *T. equiperdum* (dose 5 × 10^4^ trypanosomes/animal), became seropositive at CFT after 7–9 days p.i., while among 3 horses intravaginally infected with different doses of the parasite, only one became positive at CFT at day 23 p.i.. In a work of Hagos et al.^5^ 6 stallions were intravenously infected with *T. equiperdum* Dodola 834/940 strain (dose 50,000 parasites/ml); seroconversion was observed after 32 days p.i. by CATT/*T. evansi*.

So, just as for other pathogens, the time of seroconversion in *T. equiperdum* infections varies depending on the dose and route of inoculation, and the strain of the parasite.

Moreover, immunoblotting results obtained in the present work demonstrated that antibodies from infected rabbits, in contrast to antibodies from healthy rabbits, specifically recognise a *T. equiperdum* antigenic profile with low molecular weight bands (< 37 kDa), just as observed in dourine positive horses^22,23^. These results seem to confirm the hypothesis that *T. equiperdum* low molecular proteins are important in the humoral immunitary response against this pathogen both in horse and rabbit and could represent possible candidate diagnostic antigens for the development of more specific tests for dourine.

Some authors evaluated the cellular immune response of laboratory animals (mice, rabbits) infected with *T. cruzi* or other *Trypanosoma* species using a delayed type hypersensitivity test^30–34^. Mansfield and Kreier^33^ described positive skin test reactions of the Arthus type (type III hypersensitivity reactions) in rabbits infected with *T. congolense*. Delayed hypersensitivity reactions were observed in rabbits and mice infected with *T. brucei* and *T. rhodesiense*^27^. Thé et al.^34^ described a DTH response in a skin test performed on mice infected with *T. cruzi*. Regarding the cell-mediated response induced by *T. equiperdum* in horse, to date no scientific data are available in the literature.

Delayed hypersensitivity reactions are usually used to detect infections caused by *Mycobacterium bovis* in cattle; serological tests are used in parallel or serial testing to improve the detection of infected animals and confirm the results of intra-dermal skin tests. The use of tuberculin testing and stamping-out of reactors has eliminated *M. bovis* infection from farmed cattle in some countries^35,36^. Brucellin skin test, performed using a standardised and s-LPS-free antigen preparation, is used for the screening of unvaccinated cattle in the diagnosis of brucellosis. The brucellin skin test has high sensitivity and specificity and is also used to detect *Brucella* infections in unvaccinated sheep, goats and pigs. Moreover, the test may aid when false positive serological reactions due to cross-reacting bacteria are suspected, as false positive reacting animals always give negative results in the skin test^37^, and being independent of circulating antibodies, it might improve the diagnosis of brucellosis in latent carriers of *Brucella*^38,39^.

In this work a protein extract of OVI *T. equiperdum* was also produced and used as skin test antigen on *T. equiperdum* infected rabbits in order to evaluate its ability to produce an in vivo delayed type hypersensitivity, to obtain a diagnostic tool for the use in the field for dourine and other diseases caused by trypanosomes of the subgenus *Trypanozoon*, just as it is commonly done for brucellin and tuberculin in the detection of *Brucella* spp. and *M. bovis* infections in ruminants. Skin test antigen produced a delayed-type hypersensitivity (DHT, Type IV) response and the diameter of skin reactions obtained was in a range 8–25 mm, similarly to what can be observed in animals treated with other kind of allergens, namely tubercolin or brucellin. Moreover skin test antigen proved to be non-toxic and non-sensitizing, when tested, respectively, on guinea pigs and rabbits. These encouraging results support further studies to standardize the manufacturing process and chemical characteristics of the new skin test antigen described in this paper, in order to guarantee reproducibility of its diagnostic performances. It is also necessary to evaluate the cell-mediated response induced by *T. equiperdum* in healthy and infected horses. Since the dourine cases reported in Italy in 2011, no dourine outbreaks have been detected in Italy and in other European countries, so to date it has not been possible to test skin test antigen on naturally infected horses. However, since skin test antigens do not interfere with serological diagnosis, being independent from humoral immunitary response, they could be employed in parallel with serology to improve the evaluation of the health status of equids when dourine infection is suspected. Moreover, the lack of vaccines for dourine and surra reduces false positive results that could be obtained using skin test on vaccinated animals. Further studies should be done to evaluate diagnostic performances of skin test antigens on *T. equiperdum* infected horses in areas where dourine is endemic and to compare the cell-mediated response induced by *T. equiperdum* in both rabbits and horses. OVI *T. equiperdum* skin test antigen should also be tested on *T. evansi* infected horses to evaluate possible cross-reactions.

According to the results obtained from this study, the rabbit model could be used to increase the pathogenic knowledge of dourine, and it could help to develop more effective diagnostic tools and possibly new strategies for dourine control and prophylaxis.

## Supplementary information

Supplementary Information.
