# Supplementary material for: Humoral immune response and delayed-type hypersensitivity in rabbits infected with Trypanosoma equiperdum
Source: Sci Rep. 2020 Sep 10;10:14914. doi: 10.1038/s41598-020-71992-x (PMC7483502; doi:10.1038/s41598-020-71992-x)

**Supplemenrary material (Figures)**

**Humoral immune response and delayed-type hypersensitivity in rabbits infected with *Trypanosoma equiperdum*** (modified title)

# Tiziana Di Febo*, Ivanka Krasteva, Barbara Bonfini, Manuela Tittarelli, Osvaldo Matteucci, Gianluca Orsini, Emanuela Rossi, Michele Podaliri Vulpiani, Diamante Rodomonti, Luigi Iannetti, Mirella Luciani

Affiliation: Istituto Zooprofilattico Sperimentale dell’Abruzzo e del Molise “G. Caporale”, Via Campo Boario, 64100 Teramo, Italy

*Corresponding author

E-mail address: t.difebo@izs.it

**Supplement Figure 1.** Full-size blots of Figure 2.

After blocking, membranes were cut in strips and incubated with rabbit sera. Molecular weight marker: BenchMark Prestained Protein Ladder (bands 180, 115, 82, 64, 49, 37, 26, 19, 15, 10 kDa) (Thermo Fisher Scientific).


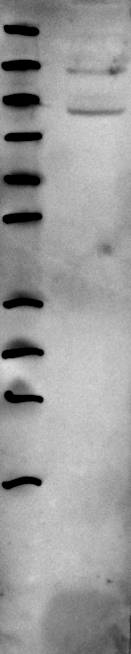

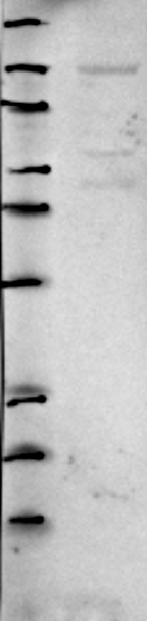

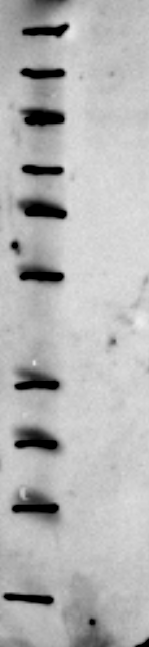

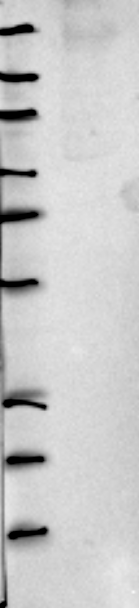

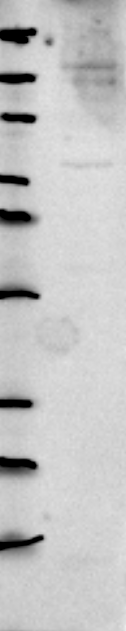


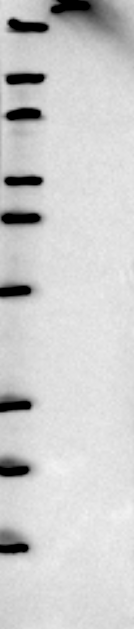

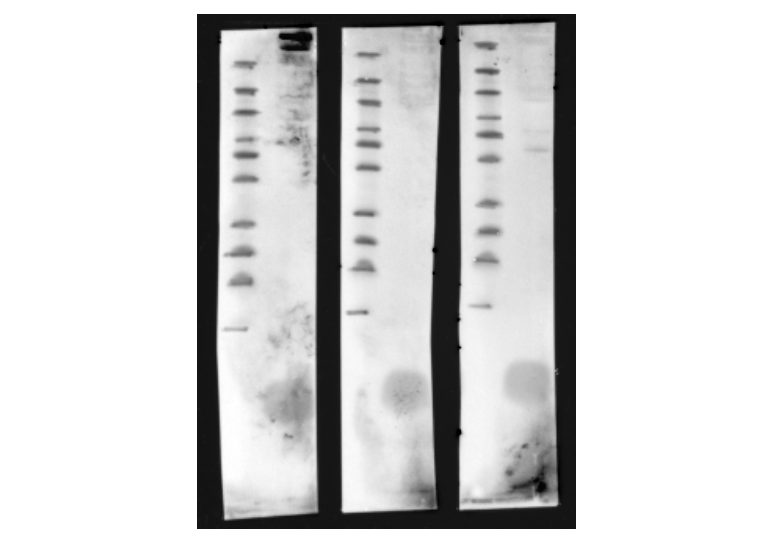

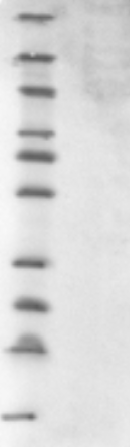

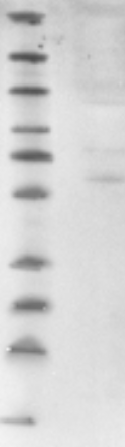


**Supplement Figure 2.** Full-size blots of Figure 3.

After blocking, membranes were cut in strips and incubated with rabbit sera. Molecular weight marker: BenchMark Prestained Protein Ladder (bands 180, 115, 82, 64, 49, 37, 26, 19, 15, 10 kDa) (Thermo Fisher Scientific).


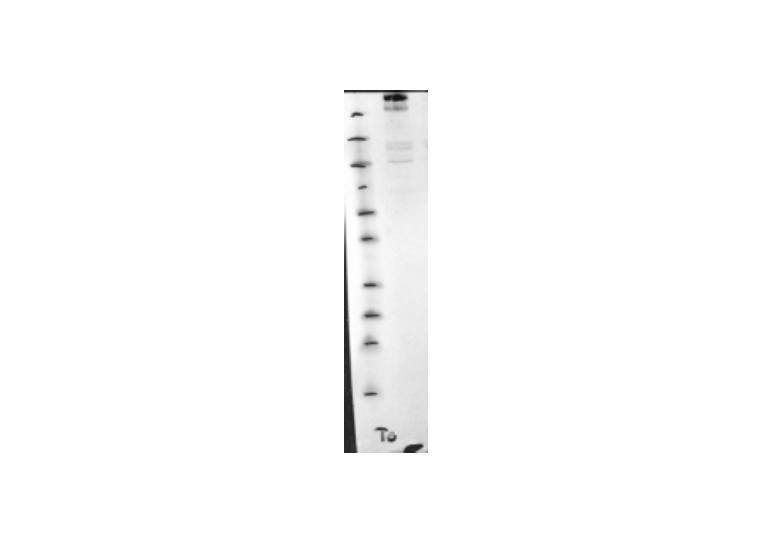

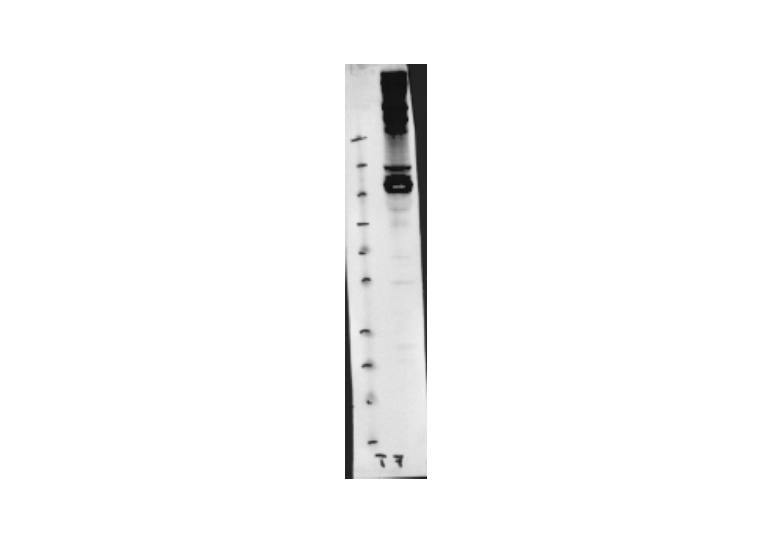

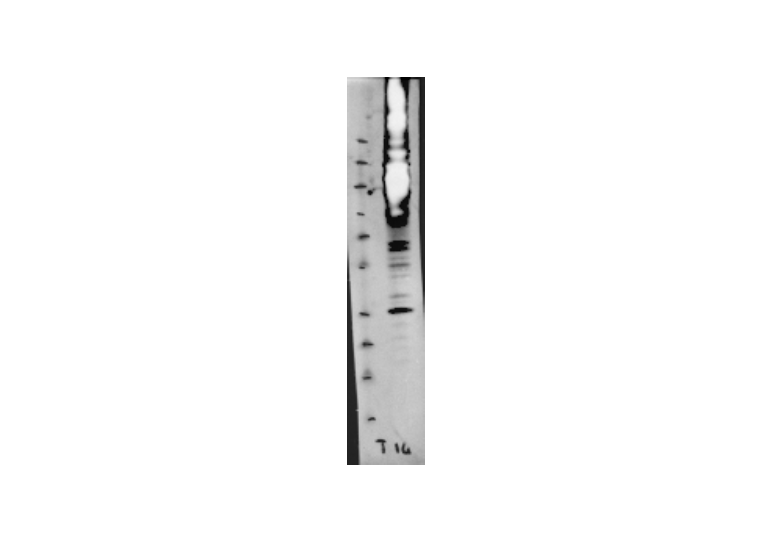

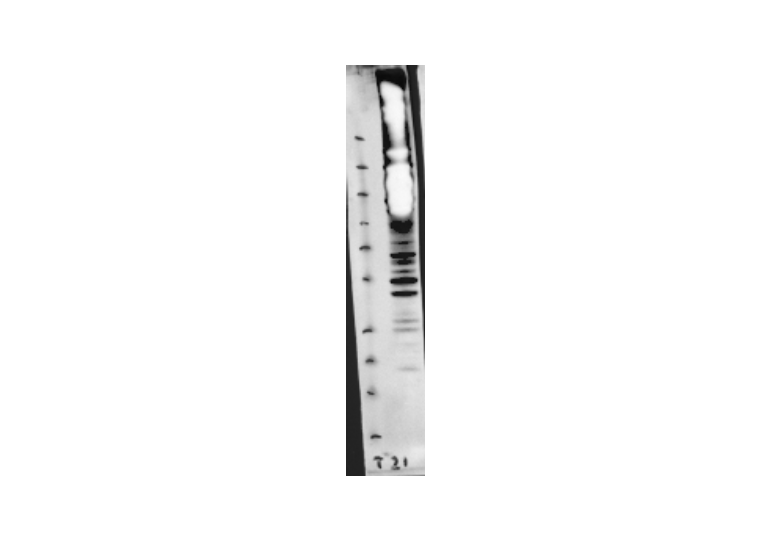

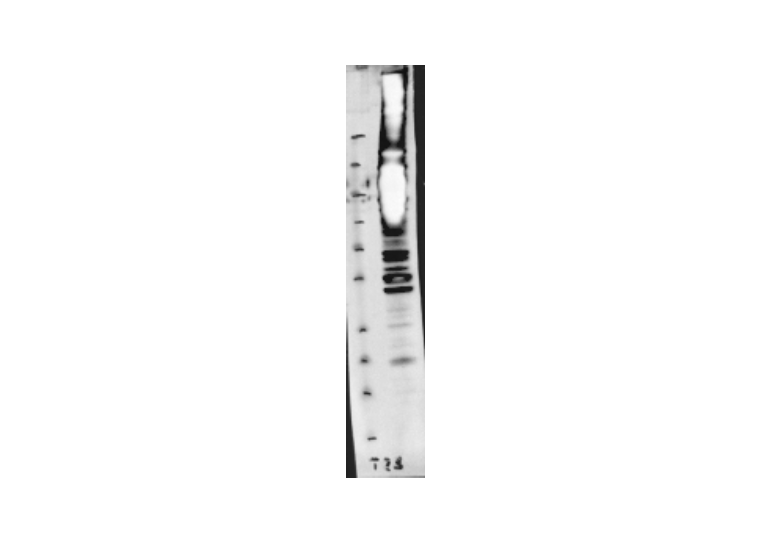

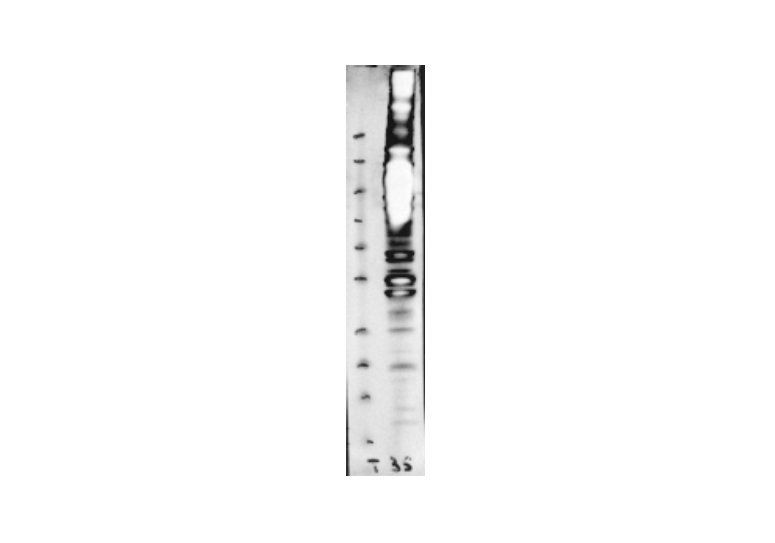

Supplement: Supplementary file 1 — Supplementary Information. [file 41598_2020_71992_MOESM1_ESM.docx]
